# Dynamic compression inhibits cytokine-mediated type II collagen degradation

**DOI:** 10.1016/j.ocarto.2022.100292

**Published:** 2022-06-30

**Authors:** Amalie Engstrøm, Frederik S. Gillesberg, Anne-Christine Bay Jensen, Morten A. Karsdal, Christian S. Thudium

**Affiliations:** a Immunoscience, Nordic Bioscience, Herlev, Denmark; b Faculty of Health and Medical Sciences, University of Copenhagen, Copenhagen, Denmark; c University of Copenhagen, Copenhagen, Denmark

**Keywords:** Asteoarthritis, Articular cartilage, Dynamic mechanical compression, Bovine explants, Type II collagen, Translational research

## Abstract

**Objective:**

To examine the effect of dynamic compressive loading applied intermittently on bovine cartilage explants stimulated with proinflammatory cytokines over 21 days.

**Design:**

Cartilage explants were cultured for 21 days with Oncostatin M and TNFα (O ​+ ​T) [10/5 ​ng/mL] or in culture medium alone (w/o). The explants were either left free-swelling or subjected to dynamic compressive loading of 20 ​min, at 1 ​Hz, with loads ranging between 0.1 and 1 ​MPa, 5 times weekly. Metabolic activity was measured once weekly using Alamar Blue and cartilage turnover was assessed with biomarkers targeting degradation fragments of aggrecan (AGNx1) and type II collagen (C2M). Glycosaminoglycan degradation was quantified was the DMMB assay. Furthermore, explant weight and histological analysis was used to assess the cartilage degradation.

**Results:**

Dynamic compression of cartilage explants attenuated the O ​+ ​T-mediated C2M release on day 21 with 40% (p ​= ​0.0068) compared to the unloaded explants. Additionally, the change in explant weight from day −1 to day 21 showed that O ​+ ​T stimulation alone mediated a cartilage loss of 11%, whereas O ​+ ​T-stimulated explants subjected to compressive loading demonstrated a decreased cartilage weight loss of 6%, which was supported by the histological analysis. However, loading had no effect on aggrecan degradation.

**Conclusion:**

In cartilage explants cultured in a proinflammatory milieu, dynamic compressive loading confers anti-catabolic effects, inhibiting type II collagen degradation and reducing explant cartilage loss. These results demonstrate that compressive loading alters cartilage tissue turnover and enforces the need to include mechanical loading in a translation *ex vivo* cartilage model.

## 1 Introduction

Osteoarthritis (OA) is a disabling disease characterized by deterioration of articular cartilage in the affected synovial joint [1]. As is true for most tissues, a healthy cartilage homeostasis is upheld by a balance of anabolic and catabolic processes, and an early hallmark of disease is the skewedness of this balance in either direction. In OA the catabolic processes dominate, especially in the cartilage tissue [2]. Unfortunately, cartilage is known to have very slow turnover, which is especially true for type II collagen which has an estimated biological half-life of more than 100 years and low potential for new formation [3,4]. Consequently, it is essential for OA patients to protect the remaining cartilage tissue by halting or slowing the ongoing degenerative processes.

It is now generally accepted that OA patients presents with chronic low-grade inflammation in the affected joint(s) [5]. However, the disease is highly heterogeneous in nature and patients can be divided into several different phenotypes of which different drivers of disease dominates, including an inflammation-driven phenotype [5,6]. Thus, while inflammation seems to play a larger role in a subgroup of OA patients, infiltration of immune cells and increased levels of proinflammatory cytokines in the joint space appears to be true for most patients in various degrees [7]. The most abundant proinflammatory cytokines present in the synovial fluid of OA patients are Tumor Necrosis factor α (TNFα), Interleukin (IL)-1β, IL-6, IL-17, and Oncostatin M (OSM), a IL-6 family cytokine [8,9]. They play a large role in cartilage destruction by inhibiting the synthesis of extracellular matrix (ECM) components and simultaneously promoting the release of proteolytic enzymes such as Matrix Metalloproteases (MMPs) and A Disintegrin And Metalloproteinase with Thrombosondin motifs (ADAMTs).

To date, disease modifying pharmacological treatments have failed in clinical trials and thus, no disease modifying OA drugs (DMOADs) are available. However, physical exercise is recommended as standard of care for patients to manage OA symptoms as it has repeatedly been shown to increase functionality and decrease pain in the affected joints [10,11]. This beneficial effect of exercise on the joint is supported by both *in vivo* and *ex vivo* loading studies on articular cartilage. In healthy rats subjected to daily low-intensity walking, gene-expression showed that exercise upregulated expression of genes required for ECM biosynthesis and suppressed genes for MMPs and ADAMTs [12]. Similarly, in a rat model of knee OA exercise 3 days weekly over 8 weeks demonstrated decreased IL-1 and MMP13 expression [13]. Torzilli et al. showed that in bovine cartilage explants stimulated with IL-1, loading over 3 days inhibited aggrecan loss, and a comparable model of bovine explants demonstrated that dynamic compression halted the catabolic response induced by a combination of joint injury and TNFα/IL-6 stimulation [14,15].

To further expand our understanding of loading on articular cartilage in response to catabolic stimuli, we developed an *ex vivo* model of compressive loading in bovine explants under inflammatory conditions. Biomarkers of cartilage tissue turnover were used to profile the structural response. Type II collagen and aggrecan as the main components of cartilage was targeted through neo-epitope biomarkers C2M and AGNx1, which measures MMP-mediated and ADAMTs-mediated degradation, respectively. These degradation markers have been quantified in both human OA cartilage explants and bovine explants, which is an advantage when working with translational cartilage models [[16], [17], [18]].

We hypothesized that dynamic intermittent compressive load confers anti-catabolic effects on cartilage tissue turnover. Hence, the objective of this study was to examine the effect of compression on bovine cartilage explants cultured long-term in presence of TNFα and OSM. We quantified the compressive loading response with the C2M and AGNx1 biomarkers, which was further supported by measurements of changes in explant weight and histological analysis.

## 2 Methods

### 2.1 Full depth articular cartilage explants

For each study, bovine cartilage explants were isolated from the hind knee of a 1–2 years old cow collected from the local slaughterhouse (Harald Hansens Eftf. I/S, Slangerup, Denmark) 24 ​h post-mortem. Using scalpel and biopsy puncher, 3 ​mm full depth cartilage disks of the load-bearing condyle were extracted from the subchondral bone. The explants were immediately transferred to and washed in Dulbecco's Modified Eagle Medium (DMEM)/F12-GlutaMAX™ (Invitrogen, MA, USA) with 1% Penicillin Streptomycin (Sigma-Aldrich, MA, USA) (culture medium). The explants were incubated overnight at 37 ​°C with 5% CO_2_ in culture medium with 2.5 ​μg/mL Fungizone (Amphotericin B, Sigma-Aldrich) to inhibit fungal infection. The explants were then cultured for 3–5 weeks in 24-well plates in either culture medium alone (w/o) or with a combination of Oncostatin M and TNFα (O ​+ ​T) (Sigma-Aldrich, MA, USA). The selection of O ​+ ​T was chosen based on previous studies of bovine cartilage explants where this combination showed to induced catabolic response [17,18]. Three times a week the conditioned medium was collected and fresh culture medium and O ​+ ​T treatment was added to the explants. The collected medium was frozen at −20 ​°C and stored for use in biomarker analysis. To examine changes in the explant weight over the experiment period the wet weight of the explants was registered on the first and last experiment day. Each explant was gently dried off to remove any adherent culture medium and placed on a laboratory scale in a sterile measuring boat. The explant weight change from baseline was calculated.

### 2.2 Intermittent compression

Compression was applied to the explants using the Electroforce® 5500 (TA instruments, Delaware, USA) with the included 24-well loading fixture, in which an individual loading pin is fitted to each explant in the 24-well culture plate allowing compressive load to be applied to 24 explants simultaneously. In each well, a thin polycarbonate washer (RH Nuttall, Birmingham, England) kept the explant in the center of the well, thus, ensuring that the explant was placed under the compressive pin of the loading fixture. The washers had an inner diameter of 5 ​mm and the explants were of 3 ​mm diameter, thus, the explants were kept in the center of the wells but unconfined. Explants were subjected to compressive loading three or five times a week depending on the study. Prior to each run, the culture plate was inserted and fastened to the loading fixture in a laminar flow hood and hereafter placed in the Electroforce® 5500 machine which was installed in a 37 ​°C incubator with 5% CO_2_. For all experiments, compression was applied in a sine wave, in which the compressive force applied to the cartilage explants cycled between 0.1 ​MPa and 1 ​MPa. Thus, within each cycle the applied force reached its peak of 1 ​MPa and its valley of 0.1 ​MPa. Each run consisted of 1200 cycles performed at 1 ​Hz (Hz) frequency. The compression run was designed and monitored using the WinTest® Software (TA instruments, Delaware, USA). After each compression run, the loading platens were autoclaved to prevents infection in the tissue cultures. This loading procedure was similarly applied in a previous study investigating the anabolic effect of compressive loading [19].

### 2.3 Studies

Three independent studies were performed with slightly different experimental setup on cartilage explants extracted from 3 different cows. A pilot study was performed with explants cultured w/o or in O ​+ ​T [10/2 ​ng/mL] over five weeks and for each treatment group six explants were applied compression three times a week and six explants remained unloaded. After 5 weeks of culture, the explants were fixed in 4% formaldehyde for 2 ​h in preparation for histology. Next, a titration study of the TNFα concentration was completed. Explants were cultured over 21 days in O ​+ ​T [10/10, 10/5 or 10/2.5 ​mg/mL] or w/o treatment. As for the pilot study, six explant per treatment group were compressed and six explants were left unloaded. Finally, the study presented here, was performed over 21 days with explants cultured w/o or in O ​+ ​T [10/5 ​ng/mL]. For each treatment group, 12 explants were subjected to compression five times a week and 12 explants remained unloaded. The frequency of weekly compression runs was increased from 3 to 5 based on optimizations studies of the model showing a larger effect of compression on cartilage biomarkers in the explants subjected to compression 5 times per week (results not included).

### 2.4 Alamar blue assay

On the first experiment day and thereafter once weekly, metabolic activity in each explant was assessed using the Alamar Blue assay (Invitrogen, MA, USA). The explants were incubated with 10% Alamar Blue in culture medium for 3 ​h, 37 ​°C, 5% CO_2_. The oxidized Alamar Blue medium was transferred to black microtiter plates and fluorescence at 540 ​nm and 590 ​nm wavelength measured excitation and emission, respectively. Four control wells without tissue were also measured and used for background-adjustment. Before applying new treatments, the explants were washed twice in culture medium and incubated in culture medium with 2.5 ​μg/mL Fungizone for 30 ​min.

### 2.5 Enzyme-linked immunosorbent assays

To examine cartilage ECM turnover in response to O ​+ ​T treatment and compression, two neo-epitope biomarkers (C2M, AGNx1) were measured in the conditioned culture medium with enzyme-linked immunosorbent assays (ELISA). The competitive ELISA C2M (Nordic Bioscience, Herlev, Denmark) was measured to quantify MMP-mediated type II collagen degradation. The assay targets the neo-epitope fragment ((KPPGRDGAAG^1053^) released from the peptide C-terminal [20]. The AGNx1 competitive ELISA (Nordic Bioscience, Herlev, Denmark) recognizes the C-terminal peptide (NITEGE^373^) released by the aggrecanases ADAMTS-4 and -5 from the aggrecan G1 domain and thus, measures aggrecan degradation [21]. For the two ELISA assays the biomarker concentration was calculated from a standard curve included on each plate measured. For samples calculated to be below the measuring range the concentration was set to the lower limit of measuring range for the assay, which was C2M: 0.13 ​ng/mL, AGNx1:1.96 ​ng/mL.

### 2.6 1.9-dimethylmethylene blue dye assay

The total glycosaminoglycan (GAG) released from the explants was measured with the 1.9-dimethylmethylene blue (DMMB) dye assay [22,23]. The conditioned medium was plated in a 96-well flat-bottom plate alongside a two-fold dilution of chondroitin sulphate A used to fit a standard curve of GAG concentration. The DMMB solution was added to the plate and the absorbance was read at 605 ​nm within 5 ​min of addition.

### 2.7 Histological analysis

The fixed explants from the pilot study were infiltrated with paraffin (Tissue-Tek VIP 5 Jr, Sakura, CA, USA) and then embedded in paraffin blocks prior to cutting 5 ​μm sections on a microtome (Microm HN 355/360). The cut cartilage sections were transferred to glass slides, deparaffinized and stained first with Hematoxylin solution and next Fast Green and Safranin O. The stained slides were then visualized in a microscope with attached camera at 20× magnification.

### 2.8 Statistics

The effect of compression on Alamar Blue, biomarker and DMMB assay results were analyzed using linear mixed model with O ​+ ​T treatment and culture day. The model assumed interaction between treatment and culture day. The explant replicate was included as a random effect. Post-hoc pairwise comparisons were assessed by contrast statements conditioned on culture day. Multiple pairwise comparisons were adjusted with Tukey's test to target as significance level of 5%. The model fits were plotted as mean ​± ​95% confidence interval. The effect of compression on explant weight change was tested within each treatment group with Wilcoxon rank sum test. Statistical tests were performed in R version 4.1.0 (R Foundation for Statistical Computing, Vienna, Austria) and RStudio version April 1, 1106 (RStudio, PBC, Boston, Massachusetts). The results were plotted in Graphpad Prism version. 9.1.2 (Graphpad Software, San Diego, California).

## 3 Results

### 3.1 Effect of dynamic compression on O ​+ ​T mediated C2M release

**Fig. 1:**
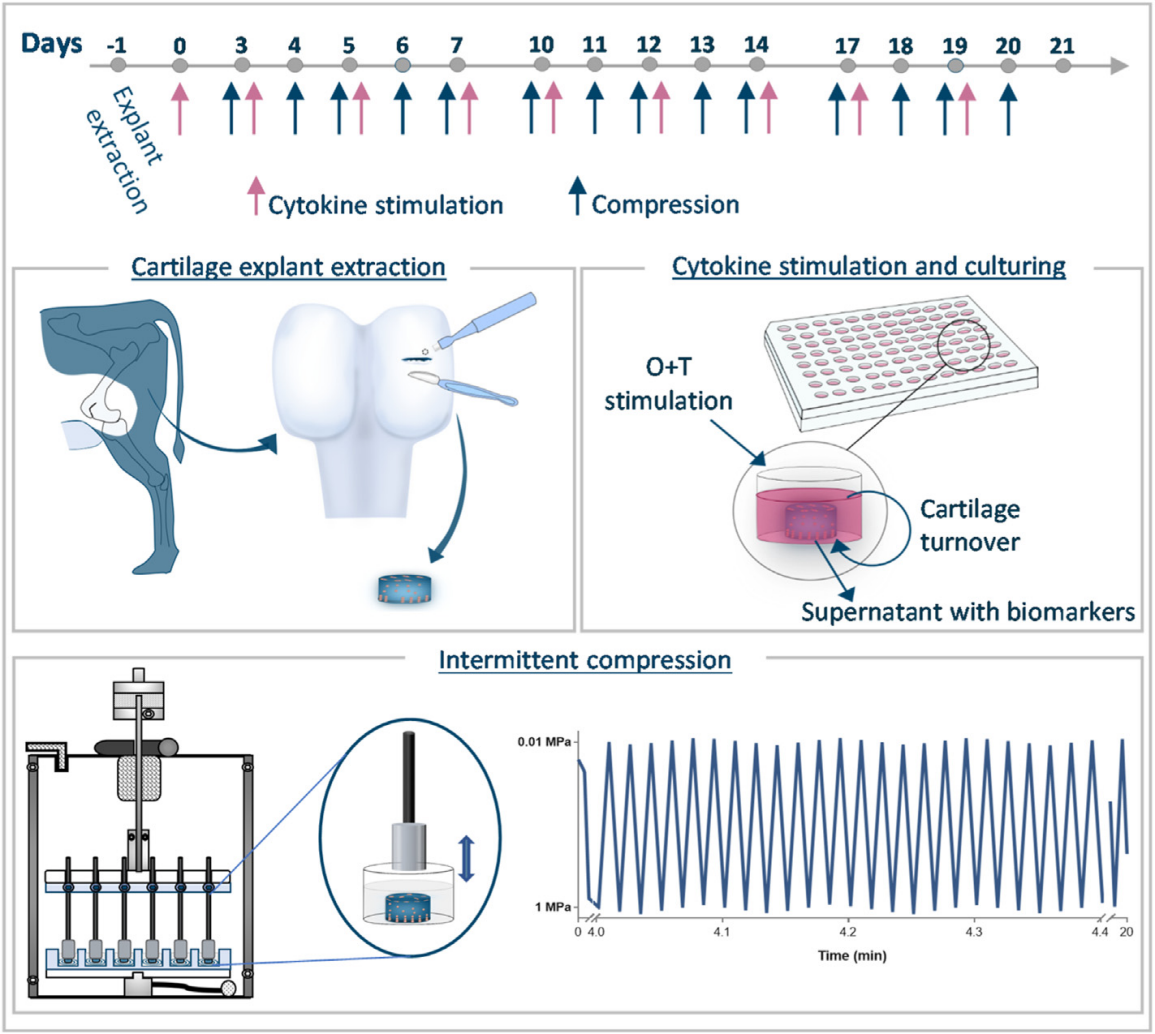
**Schematic overview of the cartilage *ex vivo* compression model. Cartilage explant extraction** (day −1). Full-depth cartilage explants are extracted from the femoral condyles of the bovine hind knee, using biopsy puncher and scalpel to release the tissue. **Cytokine stimulation and culturing** (3 days/week). The conditioned supernatant is harvested and stored at −20 ​°C for biomarker measurements. New culture medium is added with O ​+ ​T [10/5 ​ng/mL] or without (w/o). **Intermittent compression** (5 days/week). In the 24-well culture plate, the cartilage explants are fastened under individual compression pins in the loading fixture. Compression is applied in a sine wave from 0.1 to 1 ​MPa at 1 ​Hz frequency for 1200 cycles (20 ​min).

**Fig. 2:**
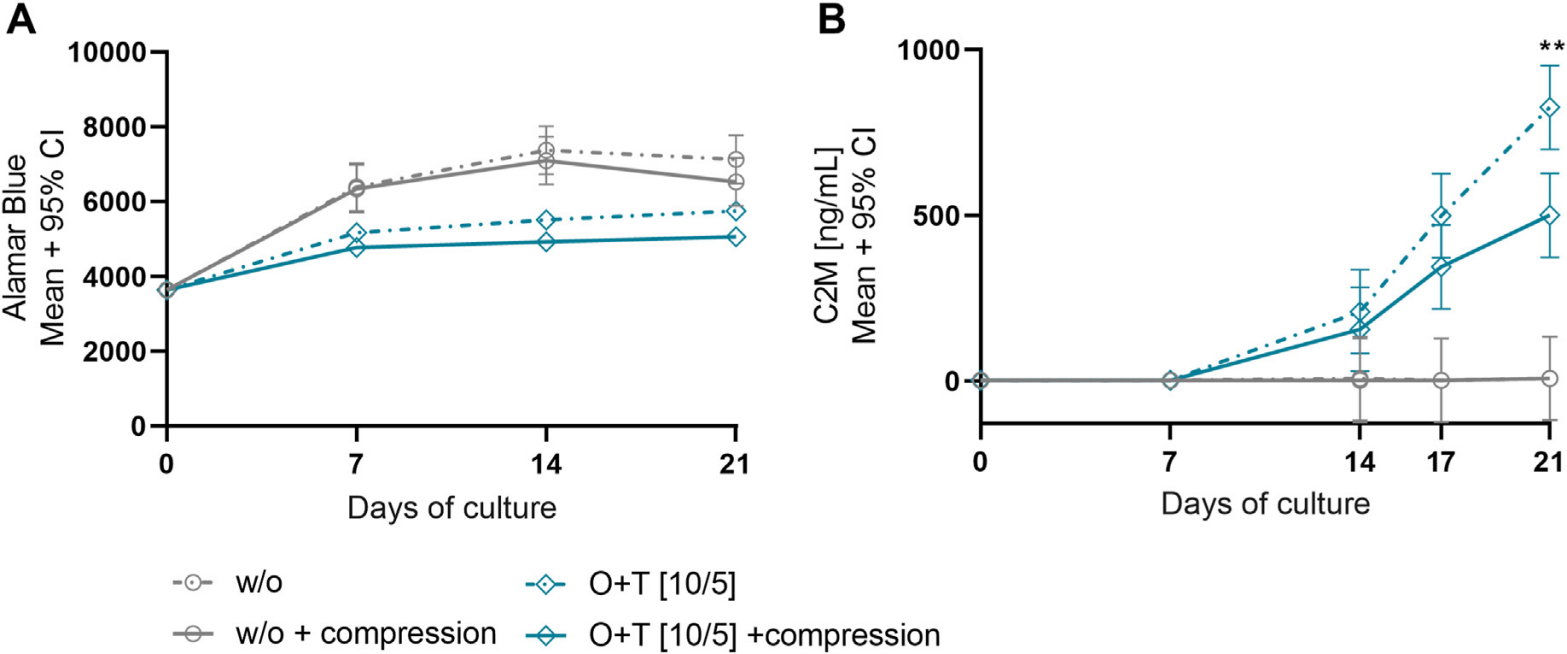
**Metabolic activity and type II collagen degradation.** Bovine cartilage explants were cultured over 21 days. Culture medium alone (w/o) or O ​+ ​T [10/5 ​ng/mL] was applied 3 times weekly and the explants were either subjected to dynamic compression 5 times weekly or left free swelling. **A.** Metabolic activity measured with the Alamar Blue assay once weekly. **B.** Type II collagen degradation measured by C2M in conditioned medium from day 0, 7, 14, 17, and 21. **A ​+ ​B.** The difference in group means was assessed with linear mixed models. Multiple pairwise comparisons were adjusted with Tukey's. The results are plotted as mean and 95% confidence interval of 12 explants and p-values are presented as: ∗∗p ​< ​0.01. (For interpretation of the references to color in this figure legend, the reader is referred to the Web version of this article.)

We aimed to test the effect of intermittent dynamic compression on O ​+ ​T-mediated cartilage degradation in bovine cartilage explants. Over 21 days cartilage explants were either cultured in culture medium alone (w/o) or treated with O ​+ ​T [10/5 ​ng/mL] and subjected to dynamic compression (1 ​Hz, 1200 cycles, 0.1–1 ​MPa) five times weekly or remained unloaded. Metabolic activity was assessed once weekly with the Alamar Blue assay and showed to remain stable in all groups throughout the culture period (Fig. 2A). Although the metabolic activity appears to be slightly decreased in the explants treated with O ​+ ​T. Type II collagen degradation was quantified with the C2M biomarker. From day 14 of culture the C2M release increased and continued to increase till day 21 in response to O ​+ ​T treatment (Fig. 2B). The O ​+ ​T-treated explants subjected to dynamic compression showed to decrease C2M release with 40% (95% Confidence interval (CI): −521.5, −130.6, p ​= ​0.0068) compared to the unloaded O ​+ ​T-treated explants on day 21. Thus, showing that dynamic compression inhibited O ​+ ​T-mediated type II collagen degradation (Fig. 1).

The culture period of 21 days was chosen based on the pilot study performed where explants were treated with O ​+ ​T [10/2 ​ng/mL] over 35 days, which showed that the C2M release peaked on day 21 (Supp. figure 1B). In the pilot study, dynamic compression similarly showed to reduce O ​+ ​T-mediated C2M release on day 17 (CI: −254.8, −402.7, p ​= ​0.0064) and 21 (CI: −228.1, −376, p ​= ​0.019) compared to the unloaded group (Supp. figure 1B). The O ​+ ​T concentration of 10/5 ​ng/mL was decided upon following a TNFα titration study in which O ​+ ​T concentrations of 10/10, 10/5, or 10/2.5 ​ng/mL was compared. The purpose of the titration study was to titrate the TNFα concentration which allowed for the largest biological window of compression, meaning the dose at which the greatest difference between loaded and unloaded samples is observed. Compression decreased C2M release in explants treated with O ​+ ​T [10/5 ​ng/mL] and [10/2.5 ​ng/mL], and an anti-catabolic effect of compressive loading was also observed on the explant weight change in the group treated with O ​+ ​T [10/5 ​ng/mL], but not in group treated with O ​+ ​T [10/2.5 ​ng/mL] (Supp. Figure 3B ​+ ​E). Hence, O ​+ ​T [10/5 ​ng/mL] was chosen for further studies.

**Fig. 3:**
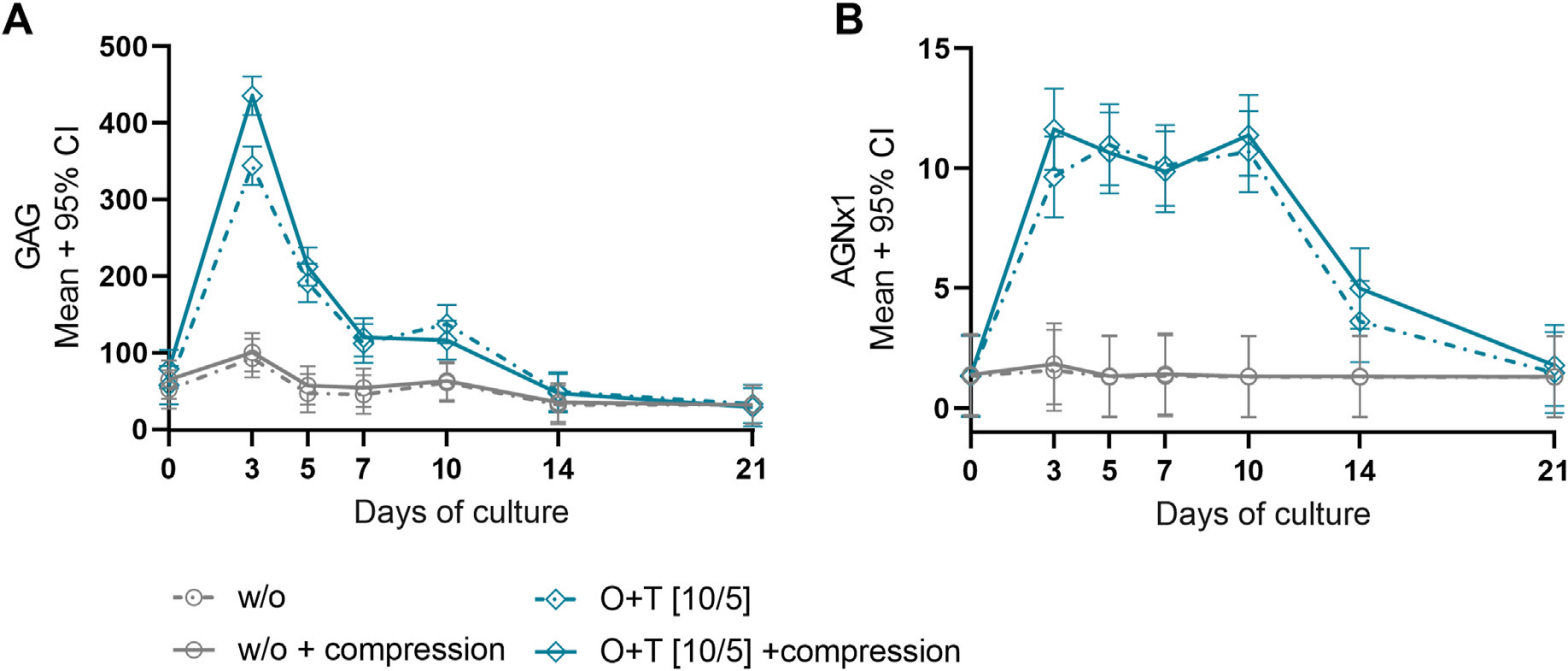
**Glycosaminoglycans and aggrecan degradation.** Bovine cartilage explants were cultured over 21 days. Culture medium alone (w/o) or O ​+ ​T [10/5 ​ng/mL] was applied 3 times weekly and the explants were either subjected to dynamic compression 5 times weekly or left free swelling. **A.** Glycosaminoglycan (GAG) release measured by the DMMB assay in the culture medium on day 0, 3, 5, 7, 10, 14, and 21. **B.** Aggrecan degradation measured by AGNx1 in conditioned medium from day 0, 3, 5, 7, 10, 14, and 21. **A ​+ ​B.** The difference in group means was assessed with linear mixed models. Multiple pairwise comparisons were adjusted with Tukey's. The results are plotted as mean and 95% confidence interval of 12 explants.

### 3.2 Effect of compression on aggrecan degradation

Applying O ​+ ​T to cartilage explants mediated extensive aggrecan degradation. The degradation was measured by the AGNx1 assay which specifically measured aggrecan degradation, and with the DMMB assay which measures the non-specific release of GAGs (Fig. 3A ​+ ​B). On day 3 the AGNx1 and GAG release was increased with 512% and 271% in the O ​+ ​T treated explants compared to the w/o group. The O ​+ ​T-mediated aggrecan degradation is further supported by histologic analysis performed on the pilot study, where the O ​+ ​T-treated explants showed a complete depletion of proteoglycans at the study termination (Supp. figure 2). The release of AGNx1 and GAG were observed to be similar in the loaded and unloaded groups, indicating that in this setup, compressive loading did not affect aggrecan degradation (Fig. 3A ​+ ​B).

### 3.3 Effect of compression on explant weight

**Fig. 4:**
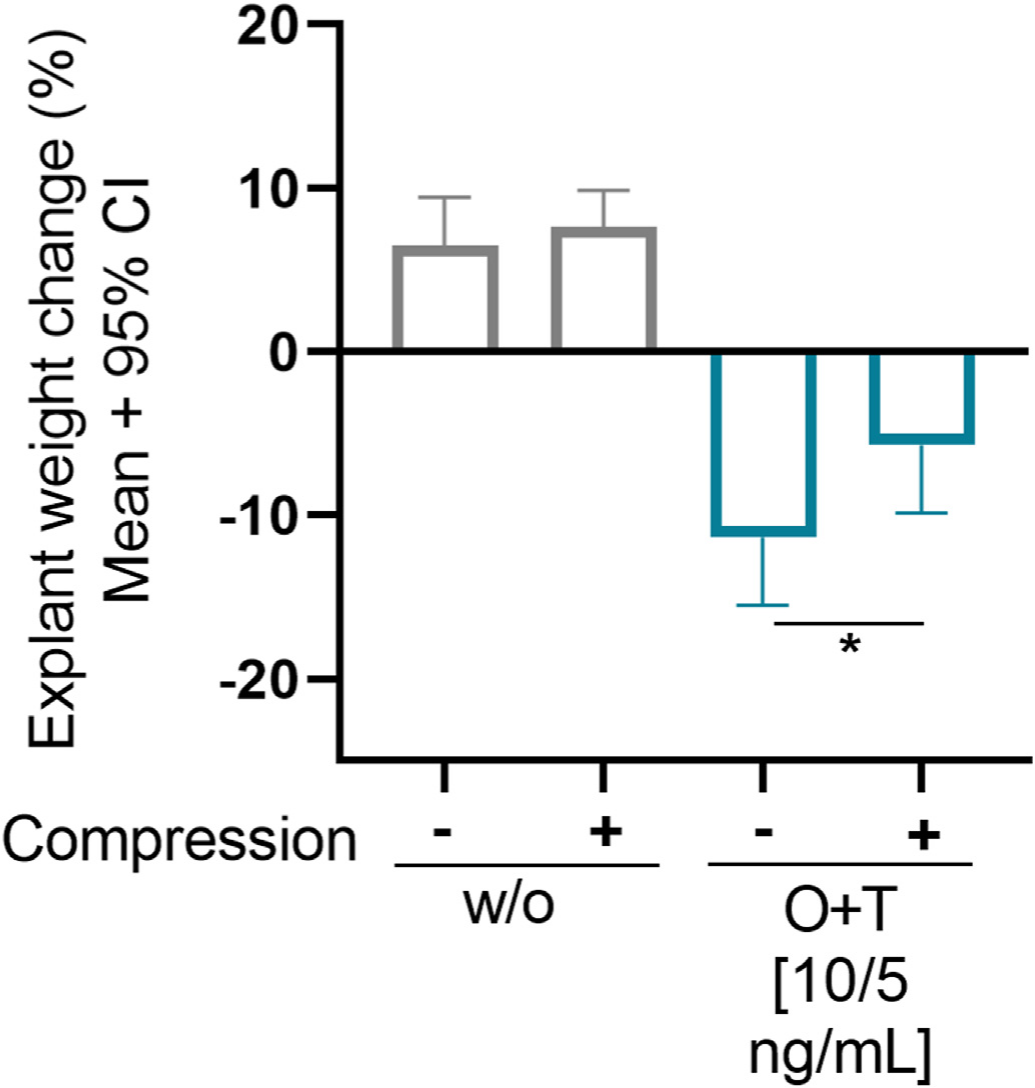
**Explant weight change from day -1 to 21.** Bovine cartilage explants were cultured over 21 days. Culture medium alone (w/o) or O ​+ ​T [10/5 ​ng/mL] was applied 3 times weekly and the explants were either subjected to dynamic compression 5 times weekly or left free swelling. Weight was assessed on day −1 and on day 21 and the percentage change in explant weight before and after the study was calculated. The difference in group means was tested with Wilcoxon rank sum test. The results are plotted as mean and 95% confidence interval of 12 explants and p-values are presented as: ∗p ​< ​0.05.

During the culture period it became apparent that O ​+ ​T treatment resulted in visible cartilage degradation, especially in the unloaded group. Thus, on the final experiment day, the explant weight change from baseline was calculated. O ​+ ​T treatment resulted in an overall explant weight loss of −11% in the unloaded group, whereas the explants cultured in culture medium alone in contrast showed an explant weight gain of 7% (Fig. 4). O ​+ ​T treated explants subjected to compression had a mean weight loss of −6%. Thus, applying compression attenuated the weight loss caused by O ​+ ​T treatment (CI: −11.8, −0.3, p ​= ​0.045). The ability of compression to inhibit this O ​+ ​T-mediated cartilage degradation was also visualized by the histologic analysis performed in the pilot study, where the unloaded O ​+ ​T treated group appeared substantially degraded compared to the compressed group, with a collapse of regular cartilage structure (Supp. figure 2). Hence, the O ​+ ​T-treated explants subjected to dynamic compression seem to preserve more of the cartilage structure than the unloaded group.

## 4 Discussion

The data presented here demonstrate that intermittent dynamic compression substantially altered the structural changes induced by proinflammatory cytokines in bovine cartilage explants. From day 14 of culture, where C2M levels began to increase, compression inhibited the O ​+ ​T-mediated type II collagen degradation. These results were supported by histological assessment of the cartilage deterioration and weight loss stimulated by O ​+ ​T and the cartilage protection exerted by dynamic compression.

These findings support the hypothesis that mechanical loading attenuates cytokine-induced catabolic processes in cartilage. On a chondrocyte level, these data are in line with an *in vitro* study in human chondrocytes where application of cyclic loading downregulated MMP-1 and -13 mRNA levels [24]. Additionally, rodent chondrocytes stimulated with IL-1B responded to dynamic strain by suppressing the IL-1B-induced signaling pathways, thus, blocking the expression of proinflammatory genes, and inhibiting the proinflammatory block of genes required for cartilage repair and chondrocyte proliferation, indicating that mechanical loading counteracts catabolic processes across several pathways [25,26].

Surprisingly, we observed no effect of compressive loading on aggrecan degradation and GAGs levels, which is in contrast to the previously mentioned study by Torzilli et al. where they observe suppression of IL-1-mediated GAG release [14]. However, there are experimental differences compared to the study presented here. Torzilli et al. applied Il-1b simultaneous with dynamic loading whereas in the present study we stimulate with O ​+ ​T for 3 days before applying compressive load. Aggrecan remodeling is initiated rapidly under inflammatory conditions, and it is possible that allowing initiation of aggrecan remodeling to occur prior to compressive loading is limiting the suppressive effect of load on aggrecan degradation. Interestingly, the lack of effect with compression on GAG and AGNx1 release suggests that loading did not have an effect on the overall release of cartilage-embedded fragments. However, we cannot exclude the possibility that the change in fluid flow with compression may lead to a change in the levels of released of cartilage fragments.

It is also worth noting that the three studies presented here show clear biological differences in the biomarker levels and weight change observed. In the main study, the unloaded O ​+ ​T-treated group released 500 ​ng/mL C2M at day 21 (Fig. 2B) and showed an explant weight difference at day 21 of 11% (Fig. 4). In comparison, the unloaded O ​+ ​T-treated [10/5] group in the titration study showed a C2M release at day 21 of 901 ​ng/mL (Suppl. fig. 3B) and an explant weight difference of 50% (Suppl. fig. 3E). Despite these differences, the relative effect of compressive loading is still observed. Furthermore, it should be noted that the cartilage explant weight was measured as wet weight, a method limited by the high amount of bound water in cartilage which in turn is affected by changes in cartilage tissue composition.

TNFα binds directly to receptors in the chondrocyte cell membrane, which activates downstream NF-κB and Mitogen-Activated Protein Kinase (MAPK) signaling pathwayss [8]. OSM can bind both the specific OSM receptor and the Leukemia Inhibitory Factor (LIF) receptor on the chondrocytes and is believed to mainly signal through the Janus Kinase (JAK) and Signal of Transducer And Transcription (STAT) signaling pathways [27,28]. While dynamic loading seems to act through several signaling pathways as mentioned above, it is above all the NF-κB pathway that has been associated to the loading response in cartilage [12,25,29,30]. This might indicate that the compression-mediated anti-catabolic effect observed here, is the result of inhibiting primarily the TNFα-induced response.

In the synovial knee joint, several cell types are involved in producing proinflammatory cytokines, including chondrocytes, synoviocytes, adipocytes and infiltrating immune cells [5]. In a cartilage explant setup, only chondrocytes are present to react and interact with the proinflammatory cytokines and the mechanical stimulation. However, the cartilage ECM plays a major part in the mechanosensation, especially through Fibroblast Growth Factor (FGF) 2 which is bound to perlecan and through fibronectin-bound TGF-β [31,32]. These growth factors are released from the ECM by dynamic compression and appear to confer both catabolic and anabolic effects in the cartilage tissue. The nature of the FGF2 response in chondrocytes is hypothesized to be dependent on the FGF receptor (FGFR), as activation of FGFR1 is believed to initiate tissue degradation whereas FGFR3 seems to promote tissue protection [33,34]. For TGF-β, high levels activate the deleterious Smad 1/5/8 pathway, while low levels activate the chondroprotective Smad 2/3 pathway in the chondrocytes [35]. Notably, TGF-β levels are elevated in the synovial fluids of OA joints compared to healthy joints, perhaps implicating a role of TGF-β in the OA pathophysiology. The complex nature of the cartilage response to mechanical loading highlights the necessity for continuous effort in understanding the underlying mechanisms and in incorporating this knowledge into DMOAD development.

The overall objective here is to optimize the *ex vivo* model translatability to clinical OA studies. By not including mechanical loading in a translational tissue model, we risk drawing conclusions based on insufficient evidence, however simulating the clinical setting of a human knee joint in movement is a complex task. We applied compressive load in a dynamic and intermittent fashion to mimic human movement, and cultured explants over a longer time to allow the cartilage tissue to adapt to both cytokine and mechanical stimulation. While the effect of short-term compressive dynamic loading of 24–48 ​h has been investigated previously, the prolonged effect of continuous intermittent loading on the tissue is still largely uncovered [[36], [37], [38], [39]]. The cartilage *ex vivo* model of compressive loading presented here subjects explants to dynamic compression intermittently over 21 days, which is to our knowledge substantially longer than other published *ex vivo* models of load applied on cartilage explants. As O ​+ ​T-mediated MMP expression has been found to increase after approximately 10 days of stimulation, it was essential for the model setup to span at least two weeks to allow measurement of the ensuing MMP-generated collagen degradation [40]. Additionally, previous studies have shown that MMP-mediated C2M release in response to O ​+ ​T treatment starts increasing at approximately 2 weeks and peaks around 3 weeks [17,18]. These findings correlate with the observed C2M release in the results presented here, where the C2M response to O ​+ ​T stimulation is seen after 2–3 weeks of culture, and it also explains why no C2M release is observed the first two weeks of the study.

Whether mechanical loading stimulate anti-catabolic and chondroprotective properties or cartilage degeneration and inflammation, sometimes referred to as mechanoflammation [41], might be a question of the state of the cartilage tissue being subjected to strain and the type, rate, and magnitude of loading. A stable joint with intact cartilage, menisci, and ligaments appears to be better protected from development of OA [42,43]. Whereas, acute excessive loading, prolonged hyper-physiological loading or loading applied in physiological range but in a non-physiologic manner have been implicated in cartilage degeneration [44]. In the present study the cartilage explants are subjected to a maximum of 1 ​MPa load, which is estimated to be in the lower end of physiological loading, although few studies have been performed to confirm the range of loading experienced by human cartilage [45,46]. Furthermore, uniaxial compressive load has been suggested to stimulate a more chondroprotective profile than shear stress, perhaps because shear stress simulate the loading profile of an unstable and malaligned joint [47]. Overall, the anti-catabolic and chondroprotective effects of the load applied in this *ex vivo* model may be a result of an appropriate type and magnitude of load and the use of healthy and uninjured cartilage explants, and it is therefore possible that damaged cartilage may respond less favorable to applied compressive load.

In conclusion, dynamic compression applied intermittently acts anti-catabolically by attenuating O ​+ ​T mediated type II collagen degradation and overall cartilage tissue loss in bovine cartilage explants. We show that in an *ex vivo* cartilage model, the application of intermittent compression altered the cartilage tissue turnover, enforcing compressive dynamic load as a necessary factor to consider in translational cartilage models.

## Author contributions

AE and CST designed the experimental model and method. CST, MAK, and ACBJ contributed to obtain funding. AE and FRG collected the experimental data. AE conducted that data analysis. AEN, CST, FRG, ACBJ, and MAK contributed to data analysis and interpretation. AE drafted the manuscript. Critical revision and final approval of the manuscript was done by all authors.

## Role of the funding source

AE is funded by the Danish Research Foundation, Denmark.

## Declaration of competing interest

CST, ACBJ, and MAK are full-time employees at Nordic Bioscience A/S. CST, ACBJ and MAK hold stocks in Nordic Bioscience. AEN and FRG have no conflicts to declare.

## Appendix A Supplementary data

Multimedia component 1 Pilot study. Bovine cartilage explants were cultured over 35 days. Culture medium alone (w/o) or O+T [10/2 ng/mL] was applied 3 times weekly and the explants were either subjected to dynamic compression 3 times weekly or left free swelling. A. Metabolic activity measured with the Alamar Blue assay once weekly. B. Type II collagen degradation measured by C2M in conditioned medium on day 14, 17, 21, and 24. C. Aggrecan degradation measured by AGNx1 in conditioned medium from day 3, 7, 10, and 14. D. Glycosaminoglycan (GAG) release measured by the DMMB assay in the culture medium on day 0 and then 3 times per week until day 35. E. Weight was assessed on day -1 and on day 35 and the percentage change in explant weight before and after the study was calculated. A-D. The difference in group means was assessed with linear mixed models. Multiple pairwise comparisons were adjusted with Tukey’s test. E. The difference in group means was tested with Wilcoxon rank sum test. A-E. The results are plotted as mean and 95% confidence interval of 6 explants and p-values are presented as: ∗ p < 0.05 ∗∗ p < 0.01. Multimedia component 1

Multimedia component 2 **Pilot study - Histological visualization of proteoglycan content by safranin O/Fast green staining.** Bovine full depth cartilage explants were isolated and cultured for 35 days. Culture media was changed with addition of O+T [10/2 ng/mL] treatment 3 times/week. Compression was performed 3 times/week. On day 35 explants were fixated, infiltrated with paraffin, embedded in paraffin, sliced, placed onto cover slides, and stained with hematoxylin, Safranin O and Fast green. For each treatment group a representative explant is shown. Scale bar represents 100 μm. Multimedia component 2

figs1 **Titration study.** Bovine cartilage explants were cultured over 21 days. Culture medium alone (w/o) or O+T [10/10, 10/5, 10/2.5 ng/mL] was applied 3 times weekly and the explants were either subjected to dynamic compression 3 times weekly or left free swelling. A. Metabolic activity measured with the Alamar Blue assay once weekly. B. Type II collagen degradation measured by C2M in conditioned medium on day 14, 17, 21, and 24. C. Aggrecan degradation measured by AGNx1 in conditioned medium from day 3, 7, 10, and 14. D. Glycosaminoglycan (GAG) release measured by the DMMB assay in the culture medium on day 0 and then 3 times per week until day 35. E. Weight was assessed on day -1 and on day 35 and the percentage change in explant weight before and after the study was calculated. A-D. The difference in group means was assessed with linear mixed models. Multiple pairwise comparisons were adjusted with Tukey’s test. E. The difference in group means was tested with Wilcoxon rank sum test. A-E. The results are plotted as mean and 95% confidence interval of 6 explants and p-values are presented as: ∗ p < 0.05 ∗∗ p < 0.01. figs1
