# Supplementary figures and images for: Dynamic compression inhibits cytokine-mediated type II collagen degradation
Source: Osteoarthr Cartil Open. 2022 Jun 30;4(4):100292. doi: 10.1016/j.ocarto.2022.100292 (PMC9718275; doi:10.1016/j.ocarto.2022.100292)

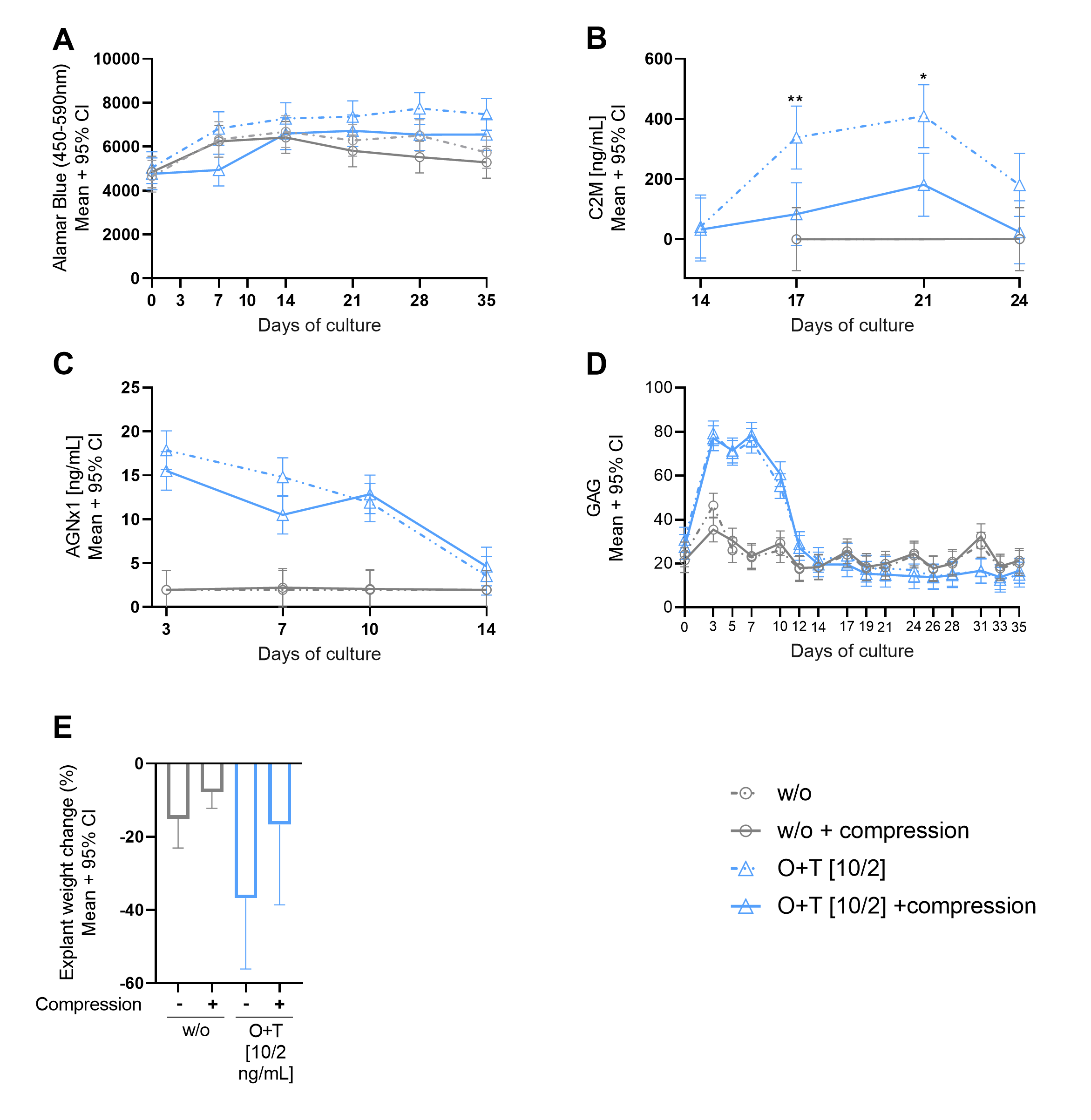

Supplement: Multimedia component 1 — Pilot study. Bovine cartilage explants were cultured over 35 days. Culture medium alone (w/o) or O+T [10/2 ng/mL] was applied 3 times weekly and the explants were either subjected to dynamic compression 3 times weekly or left free swelling. A. Metabolic activity measured with the Alamar Blue assay once weekly. B. Type II collagen degradation measured by C2M in conditioned medium on day 14, 17, 21, and 24. C. Aggrecan degradation measured by AGNx1 in conditioned medium from day 3, 7, 10, and 14. D. Glycosaminoglycan (GAG) release measured by the DMMB assay in the culture medium on day 0 and then 3 times per week until day 35. E. Weight was assessed on day -1 and on day 35 and the percentage change in explant weight before and after the study was calculated. A-D. The difference in group means was assessed with linear mixed models. Multiple pairwise comparisons were adjusted with Tukey’s test. E. The difference in group means was tested with Wilcoxon rank sum test. A-E. The results are plotted as mean and 95% confidence interval of 6 explants and p-values are presented as: ∗ p < 0.05 ∗∗ p < 0.01. [file mmc1.zip › Supp figure1.bmp]

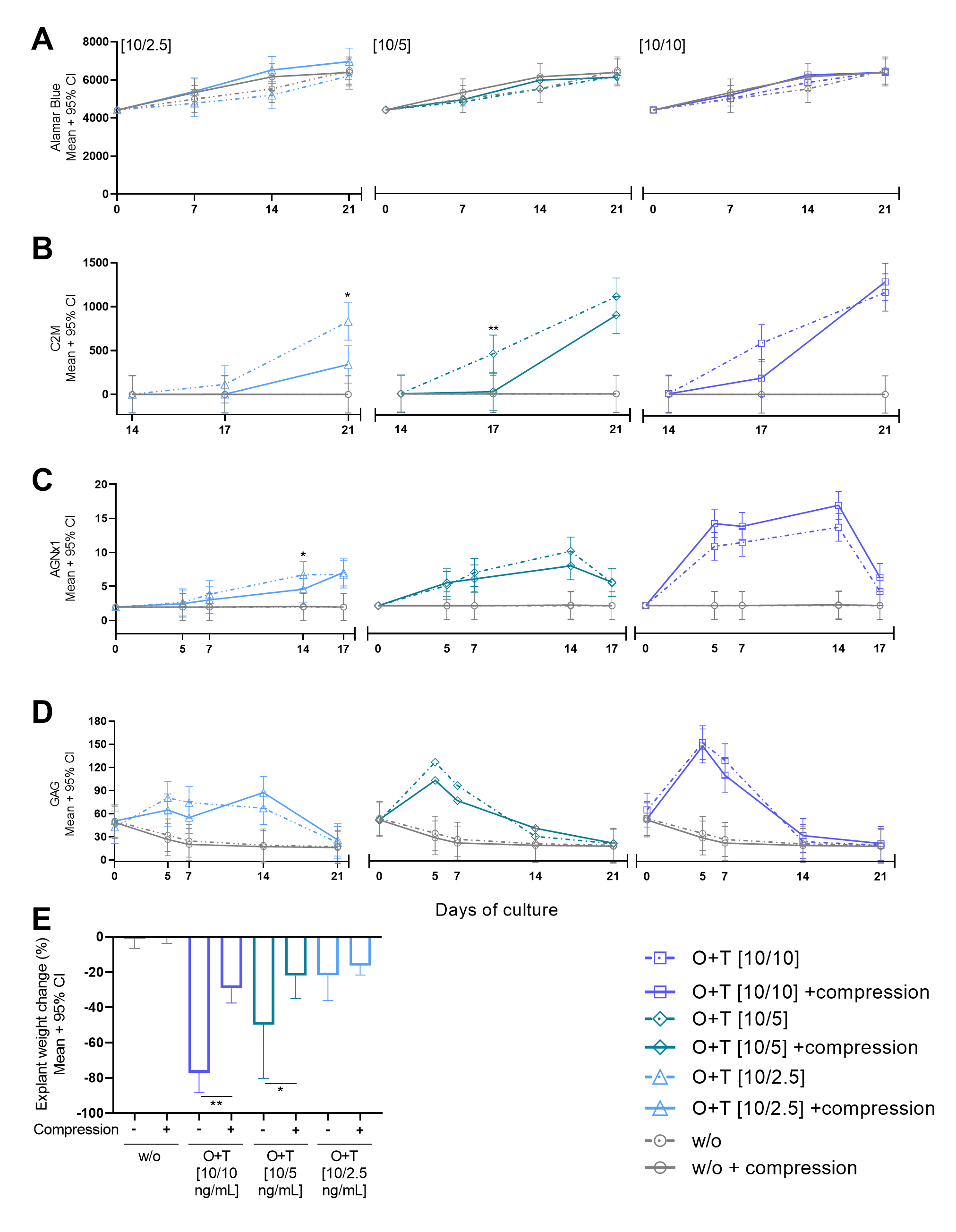

Supplement: Multimedia component 2 — Pilot study - Histological visualization of proteoglycan content by safranin O/Fast green staining. Bovine full depth cartilage explants were isolated and cultured for 35 days. Culture media was changed with addition of O+T [10/2 ng/mL] treatment 3 times/week. Compression was performed 3 times/week. On day 35 explants were fixated, infiltrated with paraffin, embedded in paraffin, sliced, placed onto cover slides, and stained with hematoxylin, Safranin O and Fast green. For each treatment group a representative explant is shown. Scale bar represents 100 μm. [file mmc2.zip › Supp figure3.bmp]

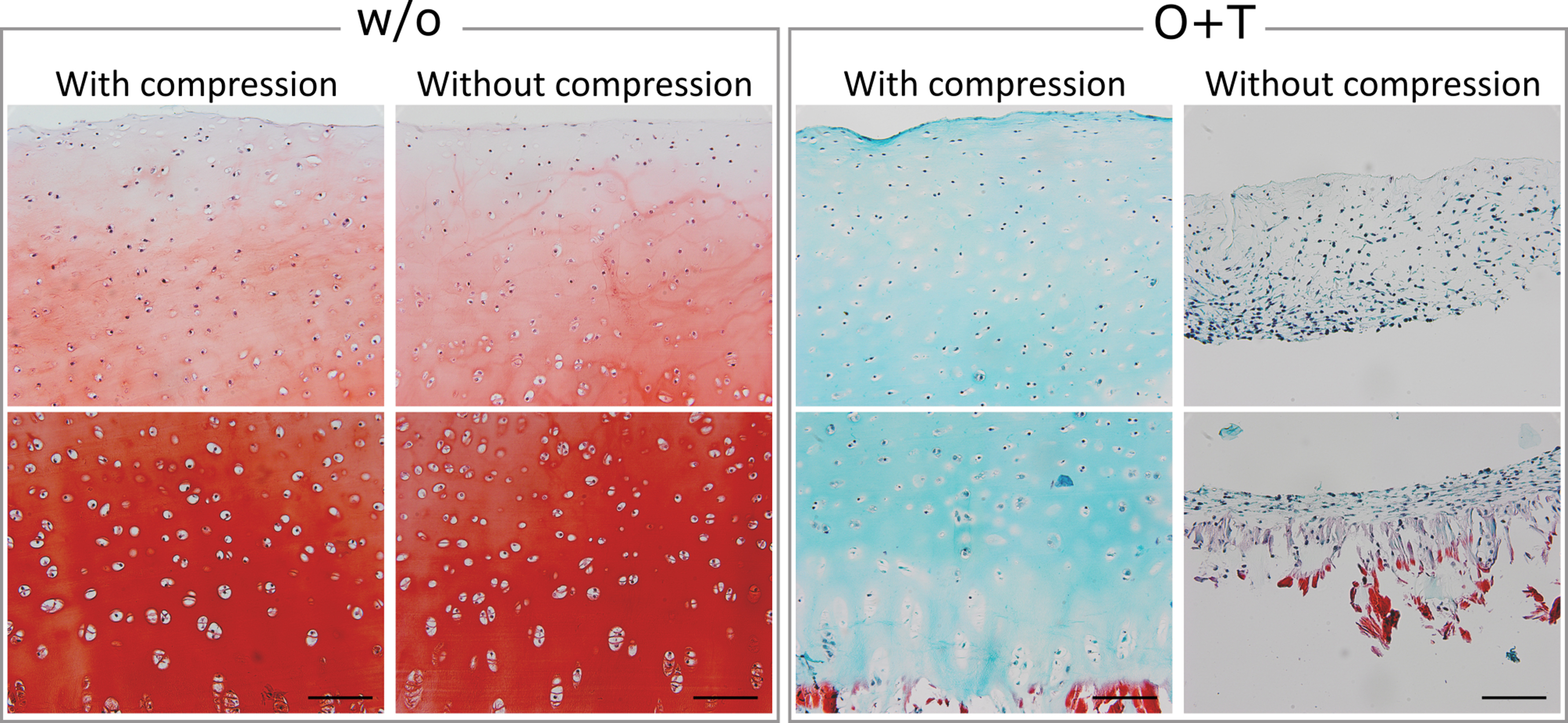

Supplement: figs1 — Titration study. Bovine cartilage explants were cultured over 21 days. Culture medium alone (w/o) or O+T [10/10, 10/5, 10/2.5 ng/mL] was applied 3 times weekly and the explants were either subjected to dynamic compression 3 times weekly or left free swelling. A. Metabolic activity measured with the Alamar Blue assay once weekly. B. Type II collagen degradation measured by C2M in conditioned medium on day 14, 17, 21, and 24. C. Aggrecan degradation measured by AGNx1 in conditioned medium from day 3, 7, 10, and 14. D. Glycosaminoglycan (GAG) release measured by the DMMB assay in the culture medium on day 0 and then 3 times per week until day 35. E. Weight was assessed on day -1 and on day 35 and the percentage change in explant weight before and after the study was calculated. A-D. The difference in group means was assessed with linear mixed models. Multiple pairwise comparisons were adjusted with Tukey’s test. E. The difference in group means was tested with Wilcoxon rank sum test. A-E. The results are plotted as mean and 95% confidence interval of 6 explants and p-values are presented as: ∗ p < 0.05 ∗∗ p < 0.01. [file figs1.jpg]
